# Role of G2-S16 Polyanionic Carbosilane Dendrimer in the Prevention of Respiratory Syncytial Virus Infection In Vitro and In Vivo in Mice

**DOI:** 10.3390/polym13132141

**Published:** 2021-06-29

**Authors:** Ignacio Rodriguez-Izquierdo, Rafael Ceña-Diez, Maria Jesús Serramia, Rosa Rodriguez-Fernández, Isidoro Martínez, Mariángeles Muñoz-Fernández

**Affiliations:** 1 Immunology Section, Head Inmuno-Biology Molecular Laboratory, Gregorio Marañón University General Hospital (HGUGM), Gregorio Marañón Health Research Institute (IiSGM), 28007 Madrid, Spain; igna.iri.93@gmail.com (I.R.-I.); rcena48@gmail.com (R.C.-D.); mjserramia.hgugm@salud.madrid.org (M.J.S.); 2 Spanish HIV HGM BioBank, C/Dr. Esquerdo 46, 28007 Madrid, Spain; 3 Hospital de Pediatría, Gregorio Marañón University General Hospital (HGUGM), Gregorio Marañón Health Research Institute (IiSGM), C/Dr. Esquerdo 46, 28007 Madrid, Spain; rrfernandez@salud.madrid.org; 4 Unidad de Infección Viral e Inmunidad, Centro Nacional de Microbiología, Instituto de Salud Carlos III, Majadahonda, 28007 Madrid, Spain; imago@isciii.es

**Keywords:** RSV infection, G2-S16 dendrimer, syncytium, nanotechnology

## Abstract

The respiratory syncytial virus (RSV) causes respiratory infection and bronchiolitis, requiring hospitalization mainly in infants. The interaction between RSV, envelope glycoproteins G and F, and cell surface heparan sulfate proteoglycans (HSPG) is required for binding and entry into the host cells. A G2-S16 polyanionic carbosilane dendrimer was identified as a possible RSV inhibitor. We speculated that the G2-S16 dendrimer adheres to the host cell-surface HSPG, acts through binding to HS receptors, and prevents further RSV infection. The G2-S16 dendrimer was non-toxic when applied intranasally to Balb/c mice, and interestingly enough, this G2-S16 dendrimer inhibits 85% RSV. Therefore, our G2-S16 dendrimer could be a candidate for developing a new possible therapy against RSV infection.

## 1. Introduction

Nanotechnology plays an important role in the treatment of broad viral infections. Particularly, polyanionic carbosilane dendrimers, a class of hyper-branched polymers with a nanoscale globular shape, well-defined functional groups at periphery, hydrophobic, or hydrophilic cavities in the interior, and low polydispersity have proven to be broad-spectrum antivirals with promising results. Globally, RSV infection is estimated at 64 million cases and 160,000 deaths annually [1]. Current advances in the use of nanoparticle technology such as dendrimers have found new opportunities that address some limitations in effective therapeutic agents. This RSV represents an important health problem in newborns, infants, young children, immunocompromised patients, and the elderly, due to the fact that there is no licensed vaccine for RSV and an effective therapeutic agent is needed [2,3,4]. RSV is the main cause of mortality and morbidity in infants until 2 years old [5].

An early RSV infection treatment is mandatory to avoid lung tissue damage, the potential development of reactive airway disorders, and complications from sustained oxygen deprivation. Although different vaccines are being studied for respiratory viruses [6], nanotechnology and different nanosystems such as gold nanomaterials have shown low toxicity and a wide range of biomedical applications, such as effective therapies [7]. Gold nanorods are being investigated as new possible treatments of RSV and for various biomedical applications [8]. Metallic nanoparticles show antiviral activity against respiratory viruses [9]. Another study made with nanoparticles against RSV was shown to have very promising data as prophylactic or therapeutic approaches, placing nanoparticles as a very interesting and effective tool; meanwhile, the vaccine is developed [10]. Additionally, mice models had improved the present airway epithelial cells culture as well as the data obtained and mimic real respiratory infections [5,11,12].

The first infection step is mediated by the interactions of RSV envelope glycoproteins G and F and the cell surface HSPG [13,14,15,16,17,18,19,20]. Indeed, to generate a compound capable of blocking the interaction between heparan sulfate (HS) and HBD, resulting in an inhibition of infection generated by different viruses, such as RSV, would be an interesting therapeutic approach. This scenario makes the G2-S16 dendrimer an important tool for antiviral research, and the dendrimers could be a new possible effective therapeutic agent against RSV. Our main objective has been to evaluate the G2-S16 dendrimer for its possible anti-RSV activity and not only to research its mechanisms of action but also its biocompatibility in human cells lines and in BALB/c mice.

## 2. Materials and Methods

### 2.1. Dendrimers and Reagents

**Figure 1:**
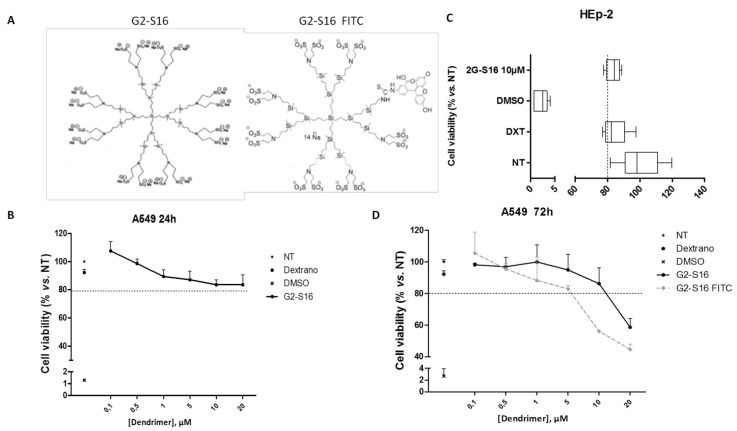
(**A**) Schematic representation of G2-S16 and G2-S16-FITC dendrimers. (**B**–**D**) Viability of A549 cells was evaluated by MTT assay after 24 h (**B**), 72 h (**D**), or 5 days of exposure (**C**) in HEp-2 cells to a range of G2-S16 dendrimer concentrations; 80% of viability was set as the limit of toxicity for the G2-S16 dendrimer. Dextran 20 μM and DMSO 10% were used as controls. Data were represented as mean ± standard deviation of three independent experiments by triplicate. NT = non-treated control; DXT = dextran; DMSO = dimethyl sulfoxide.

G2-S16 dendrimer (C_112_H_244_N_8_Na_16_O_48_S_16_Si_13_; Mw: 3717.15 g/mol) and G2-S16-FITC dendrimer labeled (C_129_H_249_N_9_Na_14_O_47_S_15_Si_13_; 3946.29 g/mol) were synthesized and characterized as previously described [21,22,23] (Figure 1A). Both dendrimers were dissolved in distillated water, and dilution to µM range was generated in distillated water from stock.

### 2.2. Cells and Viruses

The A549 human epithelial cell line (ATCC CCL-185, Manassas, VA, USA) and HEp-2 human epithelial cell line (ATCC CCL-23, Manassas, VA, USA) were grown and maintained in Dulbecco’s modified Eagle’s medium (DMEM; Biochrom AG, Berlin, Germany) supplemented with 10% heat-inactivated fetal calf serum (FBS), a cocktail of antibiotics (125 μg/mL ampicillin, 125 μg/mL cloxacillin, and 40 μg/mL gentamicin, Sigma, St Louis, MO, USA), and 1% l-glutamine in 5% CO_2_ and 37 °C.

This RSV (long Strain, ATCC VR-26) was obtained from the American Type Culture Collection (ATCC, Manassas, VA, USA) and provided by Dr. Isidoro Martinez (Centro Nacional de Microbiologia, Instituto de Salud Carlos III). This RSV was propagated in HEp-2 cells as previously described [24,25] and G2-S16 dendrimer titers were determined by plaque assay in HEp-2 cells. 

### 2.3. Screening Procedure

G2-S16 dendrimer toxicity was screened by MTT assay (Sigma, St Louis, MO, USA) according to manufacturer’s instructions. A range of concentrations between 0.1 and 20 µM were evaluated for this G2-S16 dendrimer. Dextran 10 µM and DMSO 20 µM (Honeywell, Charlotte, NC, USA) were used as control of cell viability and death, respectively, and >80% cell viability was set as the minimum non-toxicity value. A549 or HEp-2 cells were treated with G2-S16 dendrimer (0.1 to 20 µM), for 24 h in the case of A549 cells or 5 days in the case of HEp-2 cells. MTT assay was added (0.5 mg/L), and generated crystals were dissolved in DMSO. After 2 h, absorbance was read out in a Synergy 4 plate reader (BioTek, Winooski, VT, USA) at 570/690 nm.

### 2.4. Inhibition Assay

To determine the antiviral capacity of G2-S16 dendrimer against RSV, the maximum non-toxic concentration of the dendrimer was selected (10 µM). RSV infection was performed on a monolayer of A549 cells in a 6-well plate. A549 cells (5 × 10^5^) were plated and incubated for 24 h, washed with fresh DMEM medium at 2% SFB, infected with RSV at 3 Multiplicity of Infection (MOI 3), and subsequently treated with G2-S16 dendrimer. After 1 h at 37 °C, the supernatant was removed, and the infection was left in 2 mL in fresh medium at 2% FBS for 24 h at 37 °C.

Viral titration was carried out on the HEp-2 cell line. HEp-2 cells were plated in a p6, and the titration of each of the conditions was carried out, infecting with 400 µL of the viral supernatants generated in the A549 cells serially diluted (10^−1^, 10^−2^ and 10^−3^) and the adsorption for 90 min at 37 °C. After infection with the supernatants, 3 mL of DMEM 2% SFB with 0.7% low melting point agarose (LMP) were added per well and kept at 4 °C for 30 min so that the agarose solidified. The infection was maintained for 5 days at 37 °C.

Plaques of lysis generated by RSV infection were revealed by immunostaining. Cells were firstly fixed in 4% formaldehyde for 45 min at room temperature (RT); after removing the semi-solid agarose medium, the monolayer was fixed with methanol for 5 min at RT. Cells were washed twice and incubated with PBS-SAB 1% for 30 min at RT. Subsequently, the PBS-SAB was removed, and primary antibody (1:50 in 1% PBS-SAB) was added for 1 h at RT. Secondary antibody (anti-mouse-peroxidase 1:500 in PBS-SAB 1%) was added and incubated for 1 h at RT. The secondary antibody was removed, and plates were washed twice. Finally, the developing substrate was added ((citrate/phosphate 25/50 mM pH 5.0), 0.6 mL of 3-amino-9-ethylcarbazole (AEC) (Sigma, St Louis, MO, USA), 3.33 mg/mL in DMSO, and 20 µL H_2_O_2_) and after 20 min, the substrate was removed and the plates were counted. The percentage of inhibition of each condition against RSV infection was determined by comparing with controls. 

### 2.5. Inhibitions of RSV Attachment to Host Cells

The mechanism of action of G2-S16 dendrimer was determined based on the results obtained in the previous experiment, beginning to analyze the inhibition of RSV by means of the attachment mechanism, based on previously results obtained with G2-S16 dendrimer. A549 cells were plated on a p6 as described above. After 24 h, the plate was pre-cooled at 4 °C for 30 min. A549 cells were infected with RSV MOI 3 and simultaneously treated with previously selected concentration (G2-S16 10 µM) for 1 h at 4 °C. Subsequently, the cells were washed with fresh DMEM medium with 2% FBS and incubated for 24 h at 37 °C.

To titrate the RSV infection, HEp-2 cells were infected with the supernatants of the cultures obtained in the A549 cell line, according to the procedure described in the previous section, and 5 DPI, infection was revealed according to the protocol described above.

### 2.6. G2-S16 Dendrimer–Cell Interaction Assay

A549 cells were treated at maximum non-toxic concentrations of G2-S16 dendrimer for 2 h to test the capability of the G2-S16 dendrimer to interact with the cellular receptors, and it blocked the interaction of viral proteins with the host cell, inhibiting the infection. A549 cells were washed to remove G2-S16 dendrimer that did not bind to the cell surface, and MOI 3 RSV infection was performed. Then, 24 h post-infection, viral infection was revealed in HEp-2 cells as mentioned before.

### 2.7. Binding of G2-S16 Dendrimer to RSV 

To test the percentage of inhibition due to the interaction between the G2-S16 dendrimer and RSV, viral particles were pre-incubated at a MOI 3 with G2-S16 dendrimer 1 h at 4 °C. HEp-2 cells were plated in a 96-well plate in DMEM medium at 2% FBS and infected with the RSV-G2 inoculums for 1 h at 4 °C. Cells were washed twice to remove unbound virus and fixed with 4% formaldehyde and blocked with 5% SAB in PBS-Tween. Cell-bound virus was determined by incubating a primary antibody (1:50 in PBS-Tween) for 1 h at RT. Secondary antibody (anti-mouse–IgG peroxidase, 1:500 in PBS-Tween) was added after washing the plates twice, and it was incubated for 2 h at 37 °C. Then, the secondary antibody was removed, plates were washed, and ABTS developing substrate (2,2′-azino-bis- (3-ethylbenzothiazoline)-6-sulfonic acid)) (Thermo Scientific, Rock-ford, IL, USA) was added and incubated for 20 min at RT. The reaction was stopped with 1% sodium dodecyl sulfate (SDS) stop solution, and the absorbance at 405–410 nm was measured on a microplate reader BioTek Synergy™ 4 Hybrid Microplate Reader (BioTek, Winooski, VT, USA).

### 2.8. Syncytium Formation Assay

The ability of G2-S16 dendrimer to block cell-to-cell RSV spread was analyzed. A549 cell viability was determined by MTT assay at 72 h post-exposure as mentioned above. For syncytium assay, A549 cells were infected with RSV at a MOI 10 and treated with a maximum non-toxic concentration of G2-S16 dendrimer or G2-S16-FITC dendrimer immediately after RSV adsorption. Then, 72 h later, A549 cells were stained for hematoxylin/eosin (H/E) or immune-stained. For H/E stain, A549 cells were washed with PBS and stained 10 min with hematoxylin and 10 min with eosin. Syncytium formation was evaluated under light microscopy. 

Syncytium inhibition was evaluated by the immunostaining of A549 cells. Cells were infected and syncytium formation was determined by light microscopy. Once syncytia were visualized, A549 monolayer were fixed (3.7–4% PFA; 10 min), permeabilized (0.1% Triton X-100; 5 min), and incubated in PBS-BSA 5% 30 min. Monolayers were stained for RSV fusion protein (anti-human RSV, Sino Biological Inc, Beijing, China) and cellular receptor HSPG-2 (Sino Biological Inc, Beijing, China) (1:500 and 1:100 respectively). Phaloidin FITC conjugated (1:100) was used for actin filaments visualization. Secondary antibody anti-mouse IgG (Jackson InmunoResearch, Suffolk, UK) and anti-rabbit IgG (Thermo-Fisher Scientific, Waltham, MA, USA) were incubated for 1 h, nuclei were stained with DAPI, and preparations were observed in a Zeiss LSM710 confocal microscope by using Zen 2015 software (Carl Zeiss Microimaging Inc., Thornwood, NY, USA). Syncytium inhibition was determined by counting number of syncytia in random files.

### 2.9. In Vivo Assays Statement

BALB/c mice studies were approved by the CBMSO Institutional Animal Care and Use Committee (CEEA-CBMSO, Madrid, Spain) and carried out following CEEA-CBMSO, National (Royal Decree 1201/2005), according to the Directive 2010/63/EU of the European Parliament guidelines and regulations. The ability of G2-S16 dendrimer to induce irritation and damage to BALB/c mice lung when administrated intranasally was studied. Twelve BALB/c mice (Charles River Laboratories, Wilmington, MA) (22 ± 2 g and 8 weeks old) were purchased. G2-S16 dendrimer treatment was applied intranasally in BALB/c mice previously anesthetized with isoflurane (Forane, Abbott, Madrid, Spain). Therefore, BALB/c mice were randomized into four groups. The BALB/c mice control group received 50 μL of PBS intranasally. The BALB/c mice G2-S16 dendrimer group received 9.3 mg/kg, 46.5 mg/kg, or 93 mg/kg intranasally. Five days later, BALB/c mice were sacrificed, and lungs were extracted and conserved in 3.7–4% PFA *w*/*v* (PanReac AppliChem, Darmstadt, Germany). 

The possible existence of inflammation, edema, alveolar congestion, bleeding, vascular thrombosis, and congestion in pulmonary parenchyma and/or inflammatory infiltrate, edema, bleeding, fibrosis, and neovascularization in pleura were evaluated in each sample. Values (score) assigned for each of these lesions were 0–4 (no changes–very serious injuries) as previously described [7].

Histological lesions in BALB/c mice lungs were evaluated with hematoxylin-eosin staining as previously described [7]. The existence of injury in pulmonary parenchyma and pleura was evaluated in each biological sample. These values were added, and the determined level of pulmonary damage was determined as minimum (1–6), average (7–10), moderate (10–14), or severe (>14).

### 2.10. In Vivo RSV Challenge Assay

BALB/c mice were randomized in the same four experimental groups as mentioned above. All mice were intranasally infected with 2.5 × 10^6^ pfu of RSV while anesthetized with isoflurane (Forane, Abbott, Madrid, Spain). G2-S16 dendrimer was applied intranasally 2 DPI in anesthetized mice. BALB/c mice were sacrificed 5 DPI, and lungs were extracted in order to extract RSV. The viral load of each lung was determined performing serial dilutions of 5 mL of grinded RSV infected lungs and titrated on HEp-2 cells as described.

### 2.11. Statistical Analysis

Statistical study was carried out with GraphPad Prism v7.0 software (GraphPad, San Diego, CA, USA) using a *t*-Test of unpaired values and a non-parametric test, including calculation of the mean, standard deviation, and *p*-values. The significance was solved at *p* = 0.05. The data shown were analyzed from duplicate or triplicate in at least three independent experiments, unless in vivo assays, where the *n* size and the pertinent analysis were determined in its corresponding section.

## 3. Results

### 3.1. Biocompatibility of G2-S16 Polyanionic Carbosilane Dendrimer

The biocompatibility of G2-S16 and G2-S16-FITC dendrimers (Figure 1A) in A549 cells at 24 h (Figure 1B) or 72 h (Figure 1D) and in HEp-2 cells at 5 days (Figure 1C) were evaluated by an MTT assay. The G2-S16 dendrimer concentration with viability > 80% in comparison with the control group was regarded as non-toxic. The G2-S16 dendrimer was not toxic at 10 μM. Biocompatibility at 72 h post-treatment in A549 cells showed that both G2-S16 and G2-S16-FITC dendrimers were non-toxic at 5 μM.

### 3.2. G2-S16 Polyanionic Carbosilane Dendrimer against Respiratory Syncytial Virus 

**Figure 2:**
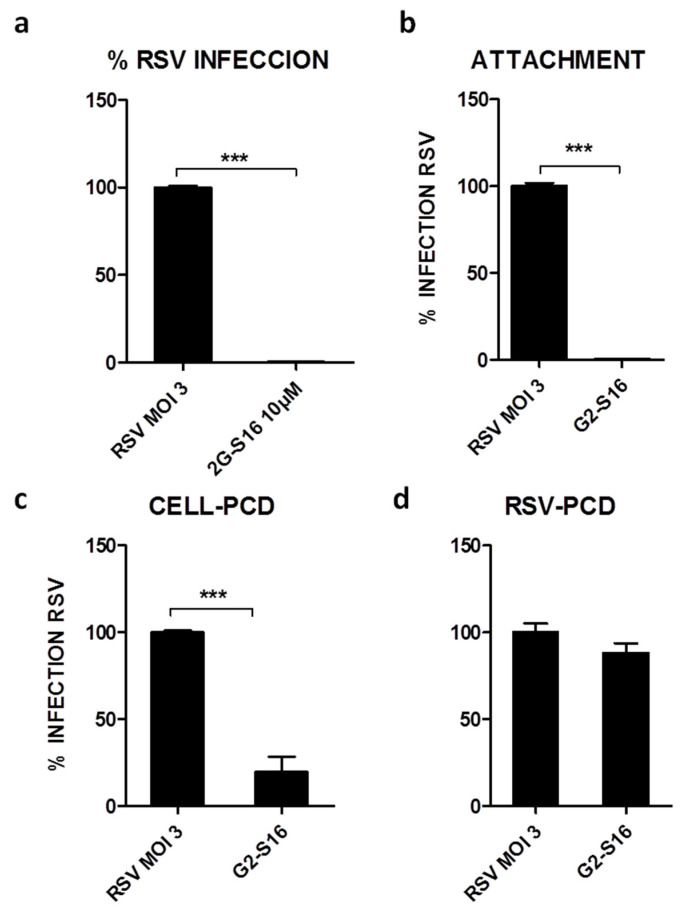
Mode of action of G2-S16 dendrimer against RSV infection. (**a**) Cells were infected 24 h, and culture supernatants were titrated in HEp-2 cells. (**b**) The effect of G2-S16 dendrimer on RSV binding. A549 cells were pre-chilled at 4 °C for 30 min and then treated and infected with RSV for 1 h at 4 °C. (**c**) Binding of G2-S16 dendrimer to cellular surface proteins. A549 cells were pre-treated 1 h with G2-S16 dendrimer. After incubation, A549 cells were washed to eliminate unbound G2-S16 dendrimer and infected with RSV. (**d**) Percentages of at one MOI 3 of RSV. The G2-S16 dendrimer was preincubated with RSV for 1 h at 4 °C. HEp-2 cells were infected 1 h at 3 MOI at 4 °C. The graph represents the relative mean of three independent experiments. G2-S16 dendrimer infection was made at a MOI 3. Infection control: non-treated infected A549 cells at 3 MOI. Data were represented as mean ± standard deviation of three independent experiments. Error bars corresponding to the SD inter-experiment (*n* = 3), *** *p* < 0.001.

We studied the G2-S16 dendrimer at maximum non-toxic concentrations for MTT assay. A549 cells were infected at an MOI 3 of RSV and treated at maximum non-toxic concentrations of G2-S16 dendrimer in Hep-2 cells. The G2-S16 dendrimer at 10 μM inhibited RSV infection by 99–100% (Figure 2A). The G2-S16 dendrimer was active at non-toxic concentrations, and this G2-S16 dendrimer halts RSV infection (*p*-value < 0.001 vs. control) in A549 cells.

### 3.3. Mechanism of Action of G2-S16 Polyanionic Carbosilane Dendrimer

The antiviral activity of G2-S16 dendrimer against RSV infection and the underlying mechanism of action was studied (Figure 2). We previously demonstrated that G2-S16 dendrimer inhibits RSV infection in a significant manner (99–100%) (Figure 2A). We previously described that G2-S16 dendrimer inhibition against other viruses occurs in the early steps of the infection, acting in attachment events due to the interactions between peripheral groups and viral or host cells proteins or receptors [26,27]. In the case of RSV, the G2-S16 dendrimer also inhibits the attachment of RSV with A549 cells (99–100%; *p*-value < 0.0001) (Figure 2B). This inhibition could be due to the interactions of the G2-S16 dendrimer either with A549 cellular entry receptors or with viral proteins. 

To test the first hypothesis, A549 cells were incubated 30 min with G2-S16 dendrimer and we washed the unbound G2-S16 dendrimer prior to RSV infection, resulting in a significant RSV inhibition of 83–84% (*p* < 0.01) (Figure 2C). The next step was to test the other interaction of the G2-S16 dendrimer with the RSV virions hypothesis. In this case, not significant inhibition was observed when the G2-S16 dendrimer was incubated with RSV particles prior to infecting A549 cells (11–12%) (Figure 2D). These results are in line with our previous experiments, demonstrating that the inhibitory action of G2-S16 dendrimer was due to an interaction with host cell receptors.

### 3.4. Inhibition of Syncytium Formation

**Figure 3:**
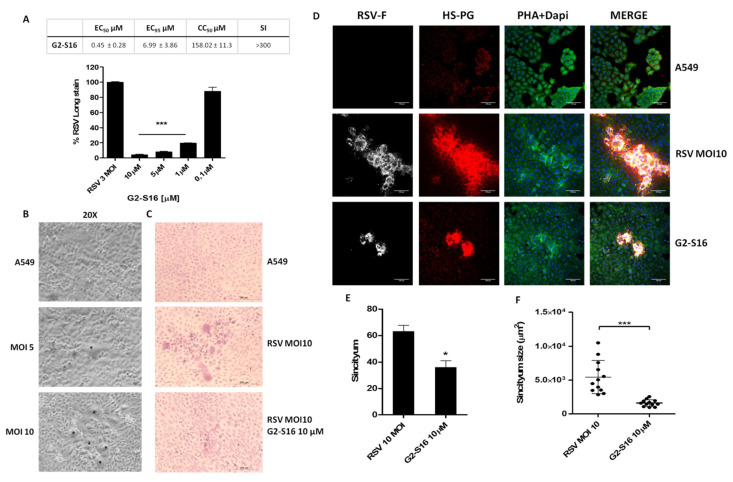
Syncytium formation assay. (**A**) A549 cells were infected at a MOI 3 to evaluate the inhibition of G2-S16 dendrimer. EC50, EC95, CC50, and SI were calculated. (**B**) A549 cells were infected at a different MOI of RSV to evaluate the syncytium formation (black asterisk). A549 cells were infected at a MOI 10 (**C**–**F**) of RSV and treated with G2-S16 dendrimer in a range of non-toxic concentrations. Then, 72 h post-infection, cells were (**C**) H/E stained or immune-stained for (**D**) RSV-F viral glycoprotein (white), HSPG (red), and actin filaments (phalloidin; green). Nuclei were stained with DAPI (blue). (**E**) Media of syncytium formation/well after 72 h post-infection. (**F**) Media of syncytium size in four random fields/experiment after 72 h post-infection analyzed with ImageJ software. Images are representative of three independent experiments. Data showed the media ± SD of three independent experiments. *: *p* < 0.05; ***: *p* < 0.001.

Syncytium formation is a well-known mechanism of cell-to-cell infection that contributes significantly to virus spread in vivo. The RSV specifically uses syncytium formation to spread infection. To determine whether G2-S16 dendrimer prevents cell-to-cell spread of RSV after infection, a syncytium formation assay was performed on infected A549 cells after 72 h. The G2-S16 dendrimer was not toxic at the range of concentrations evaluated on A549 cells (Figure 1D). The inhibition range of the G2-S16 dendrimer was also measured in A549 cells at a MOI 3 (Figure 3A) to determine EC_50_ and subsequently, therapeutic index (SI).

Firstly, A549 cells were infected with RSV at a different MOI to allow syncytium formation and visualization. MOI 10 was selected for syncytium assays (Figure 3B). A549 cells were infected with RSV, treated with G2-S16 dendrimer, and stained for H/E (Figure 3C). Even the increase in RSV quantity, the G2-S16 dendrimer was able to reduce the number and mainly the size of the syncytium generated (Figure 3E,F). Infected A549 cells were also immune-stained to confirm inhibition observed in optical microscopy (Figure 3D). A549 cells were fixed 72 h post-infection and stained for RSV-F viral protein (white), HSPG (red), phalloidin (green), and nuclei with DAPI (Figure 3D). Our data demonstrated that treatment with G2-S16 dendrimer, even RSV infection, when performed at a MOI 10 reduced significantly the syncytium quantity and mainly the size of the formed multinuclear cells. Interestingly, when RSV infection occurs and syncytium is formed, the images reveal a polarization in the main receptor of RSV, HSPG, favoring infection and co-locating with RSV-F. We demonstrated that the G2-S16 dendrimer reduces syncytium formation, particularly the size of the syncytium, indicating a promising function against RSV infection. Thus, the next step was to locate the G2-S16 dendrimer in syncytium formation.

**Figure 4:**
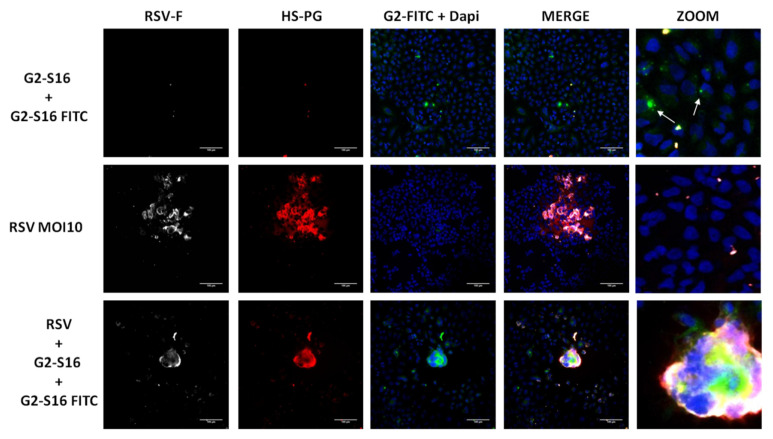
Presence of G2-S16 dendrimer in syncytium formation. A549 cells were infected with RSV at MOI 10 and treated with a mix of G2-S16 (5 µM) and G2-S16 FITC (5 µM) dendrimers. Then, 72 h post-infection, A549 cells were fixed and immune-stained for RSV-F glycoprotein (white), HSPG (red), and DAPI nuclei visualization (blue). Images are representative of three independent experiments.

The G2-S16-FITC labeled dendrimer was used to determine the position of the G2-S16 dendrimer in syncytium generation. A549 cells were infected at MOI 10 of RSV as mentioned above and treated with a mix of non-toxic concentrations (Figure 1) of G2-S16 dendrimer (5 µM) and G2-S16-FITC (5 µM) to observe visualization of the dendrimers. Then, 72 h post-infection, A549 cells were immune-stained for RSV-F glycoprotein and HSPG. G2-S16-FITC was observed in green (Figure 4). DAPI was used for nuclei visualization.

As expected, in fields without the presence of RSV, the G2-S16 dendrimer is located inside A549 cells, mainly at the cellular surface (Figure 4; white arrows). These data are in concordance with cellular protection assay results, where the G2-S16 dendrimer is able to interact with A549 cells and protect RSV infection. In these fields where syncytium was generated, the G2-S16-FITC dendrimer was visualized inside the syncytium (Figure 4), suggesting that it could interfere with adjacent cells, avoiding syncytium spread. 

### 3.5. In Vivo Experiments: Murine Lung Model for Viability of G2-S16 Dendrimer and Respiratory Syncytial Virus Challenge 

Once it was demonstrated that G2-S16 dendrimer inhibits RSV and the underlying mechanism of action by which the inhibition occurs, we studied if G2-S16 dendrimer induces irritation and/or damage in mice lungs when this G2-S16 dendrimer was administrated intranasally in BALB/c mice. No damage or alteration to the lung epithelium was shown when the G2-S16 dendrimer was applied intranasally at concentrations up to 46.5 mg/kg in mice lung. However, the G2-S16 dendrimer showed slight toxicity in lungs (inflammation, vascular thrombosis, and congestion and atelectasis) at high concentrations of 93 mg/kg. However, those data suggest that the application method could be the reason for the G2-S16 dendrimer toxicity in vivo (Table 1).

Histological analysis of mice lung clearly reveals the minimum or average damage in all analyzed lungs. This is probably due to the application method, because PBS, which is non-toxic control, also reveals average damage in all analyzed samples. 

**Figure 5:**
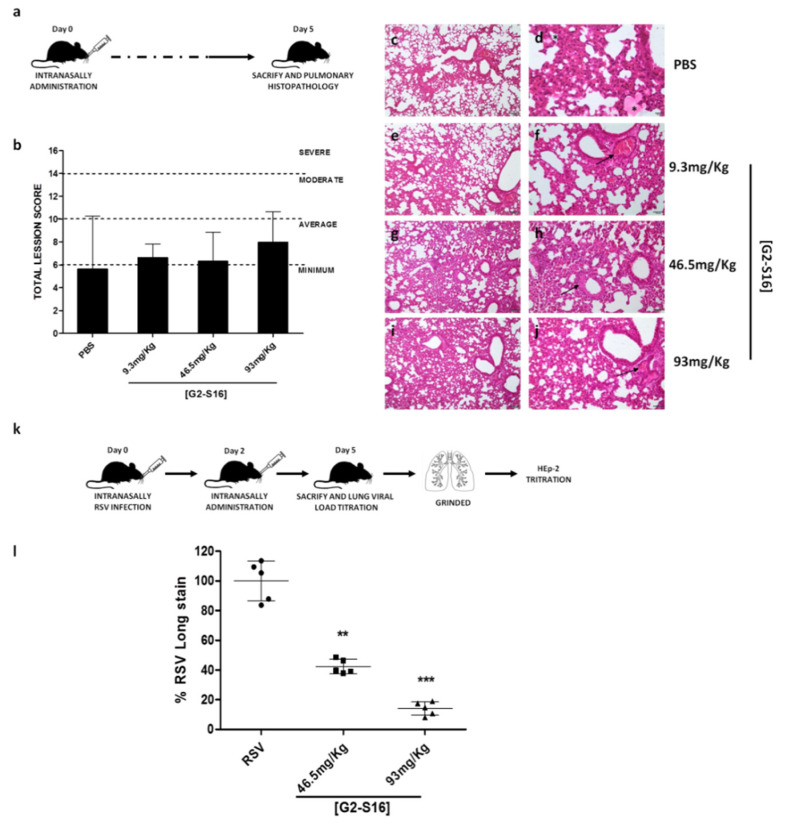
In vivo experiments with G2-S16 dendrimer. (**a**) Schematic representation of in vivo viability procedure. (**b**) Media of the accumulate score for each treatment group. (**c**–**j**) Histological studies of pulmonary damage. (**b**–**d**) Focal and minimal inflammatory infiltrate and atelectasis around the presence of an acellular and eosinophilic substance (possibly PBS) (asterisk). (**e**,**f**) Alveolar congestion of medium severity mainly around bronchioles and vessels in both cases (arrows). (**g**,**h**) Thickening of the walls of the arterioles in the areas of inflammation (arrows). (**i**,**j**) More diffuse lesions in the lung parenchyma with congestion and alveolar atelectasis. Presence of thrombi in vessels (arrow). (**k**) Schematic representation of in vivo antiviral activity procedure. (**l**) Percentages of inhibition of G2-S16 dendrimer corresponding to RSV infection of BALB/c mice and subsequent titer of the lung infection HEp-2 cells for 5 days. The figure represents the relative mean of five mice per group. RSV: BALB/c mice RSV infected and treated with PBS. **: *p* < 0.01; ***: *p* < 0.001.

Due to the data obtained in an accumulated score, the presence of pulmonary injuries in treatment with PBS showed that scores observed in G2-S16 dendrimer treatment are not significantly related to PBS biocompatibility control, suggesting that the G2-S16 dendrimer is safe for further in vivo assays (Figure 5). It is also important to note that no damage was detected in pleura analysis in any of mice analyzed samples. Mice samples treated with G2-S16 dendrimer present mainly an alveolar congestion and a moderate infiltrate, being more serious in samples treated with 1 mM of G2-S16 dendrimer and minimum in those BALB/c mice treated with 9.3 mg/Kg of G2-S16 dendrimer. Based on the data obtained, the concentration of 93 mg/Kg is the best one as well as the concentration selected for RSV in vivo challenge. 

Finally, we also studied the antiviral activity of the G2-S16 dendrimer against RSV challenge in BALB/C mice. Intranasal administration of the G2-S16 dendrimer reduced RSV replication and infection in a significant manner. The administration of 50 µL of G2-S16 dendrimer 93 mg/Kg, after 4 h post-exposure to a high dose of RSV, abrogated RSV infection in 86% (*p* < 0.0001 vs. placebo) (Figure 5). Interestingly and importantly, the G2-S16 dendrimer at 46.5 mg/Kg inhibited RSV infection in 56% of BALB/C mice. 

## 4. Discussion

RSV is the main cause of respiratory mortality and morbidity in persons of all ages [28,29,30]. Particularly, RSV is the main cause of acute lower respiratory tract infections in children less than 5 years of age and responsible for substantial disease burden in the elderly. The G2-S16 dendrimer presents different types of functionalized groups at their periphery, including antiviral activities [31,32]. The biodistribution and biocompatibility of the G2-S16 dendrimer have now been evaluated after administration at a systemic level, and resistant mutation appearance was evaluated in HIV-1 infection [33].

Herein, we demonstrate that the G2-S16 dendrimer inhibits RSV infection not only in an in vitro cellular model but also in a BALB/c mice *in vivo* model. Evaluation of the cytotoxicity of the G2-S16 dendrimer has shown that this dendrimer is non-toxic in A549 cells at selected concentrations and that this G2-S16 dendrimer is capable of significantly inhibiting RSV infection in the A549 cell line (>99%) (Figure 2). Studies of cytotoxicity and inhibition curves made it possible to calculate the selectivity index (SI) of the G2-S16 dendrimer, enabling a value of >300 for RSV inhibition. Based on these previous results, the mechanism of action by which the G2-S16 dendrimer inhibits RSV and the interaction with A549 cells was studied.

Firstly, data obtained in attachment assay demonstrated that the inhibition by G2-S16 dendrimer (99–100%) (Figure 2) was due to the ability of the G2-S16 dendrimer to destabilize or prevent the interactions of the RSV virions with the cell host receptors. This first step confirms that G2-S16 dendrimer inhibition occurs in the entry early events of RSV infection. Data also confirm that this inhibition is mediated by an interaction of G2-S16 dendrimer with cellular receptors (Figure 2); meanwhile, the interaction of the G2-S16 dendrimer with the RSV viral particles does not generate a significant effect in the RSV infection (Figure 2). All these data demonstrate that the G2-S16 dendrimer is capable of interacting with host cellular receptors implied in the RSV infection. We previously described that G2-S16 dendrimer interacts with cellular HS and HBD in HSV infection [34,35,36,37]. We also previously described that the G2-S16 dendrimer could interact electrostatically with the viral glycoproteins of the viral surface as well as cellular receptors [21,22]. Interestingly, the RSV cellular entry mediators in the viral infection are mainly the HSPG, in such a way that it allows us to hypothesize that the RSV inhibition demonstrated by the G2-S16 dendrimer could be due to the interaction of the dendrimer with HSPG of the cellular surface, blocking this crucial initial step of the RSV infection. 

In addition to the mechanism of action, we described the ability of the G2-S16 dendrimer to halt RSV infection in vitro related to the syncytium formation and size in pulmonary A549 cells (Figure 3). Our data suggest that the G2-S16 dendrimer halts syncytium formation targeting cellular receptors, which are data that are in concordance with the results obtained in mechanism of action assays.

Due to the potential use of the G2-S16 dendrimer as a possible treatment against RSV in vivo, we studied its biocompatibility in BALB/C mice. Our results clearly showed that the G2-S16 dendrimer applied intranasally at 93 mg/kg in BALB/c mice was well tolerated, and no significant signs or damage to pulmonary parenchyma or pleura were observed (Table 1). In addition, we also analyzed the antiviral activity of the G2-S16 dendrimer in BALB/c mice, and we demonstrated that it decreased by 86% when RSV infected BALB/c mice were treated with this G2-S16 dendrimer (Figure 5).

## 5. Conclusions

The G2-S16 dendrimer was demonstrated to be an effective treatment against RSV infection. G2-S16 inhibits RSV infection in vitro and in vivo, and it presents good biocompatibility in vivo. In conclusion, G2-S16 dendrimer therapeutic application needs careful design and treatment regimen to be considered effective for RSV treatment. G2-S16 dendrimer in combination with biocompatible drugs and/or molecules could be a promising therapeutic for that infection. The use of G2-S16 dendrimer could also contribute to finding a solution to prevent RSV infection in infants and other target populations. 

## Figures and Tables

**Table 1:** Lung and pleura toxicity assay. The existence of injury in pulmonary parenchyma (inflammation, edema, alveolar congestion, bleeding, and vascular thrombosis or congestion) was evaluated in each biological sample.

Pulmonary Parenchyma	PBS			9.3 mg/kg			46.5 mg/kg			93 mg/kg		
Inflammation	0 ^1^	1	3	2	2	2	2	1	3	2	2	1
Edema	0	0	0	0	0	0	0	0	0	0	0	0
Alveolar congestion	0	0	3	1	1	2	1		2	3	2	1
Bleeding	1	0	1	1	0	1	0	0	0	0	0	0
Vascular thrombosis or congestion	1	1	1	0	1	1	1	2	1	2	3	2
Atelectasis	1	1	3	2	2	2	2	1	3	3	2	1
Emphysema	0	0	0	0	0	0	0	0	0	0	0	0
**Pleura**	**PBS**			**9.3 mg/kg**			**46.5 mg/kg**			**93 mg/kg**		
Inflammatory infiltrate	0	0	0	0	0	0	0	0	0	0	0	0
Edema	0	0	0	0	0	0	0	0	0	0	0	0
Bleeding	0	0	0	0	0	0	0	0	0	0	0	0
Neovasculation	0	0	0	0	0	0	0	0	0	0	0	0

^1^ 0 (no change); 1 (minimum); 2 (light); 3 (moderate); 4 (very serious). These values were added up to determine the level of vaginal irritation as minimum 1–6, average 7–10, moderate 10–14, and severe 14+.
